# LLIN Evaluation in Uganda Project (LLINEUP): a cross-sectional survey of species diversity and insecticide resistance in 48 districts of Uganda

**DOI:** 10.1186/s13071-019-3353-7

**Published:** 2019-03-12

**Authors:** Amy Lynd, Samuel Gonahasa, Sarah G. Staedke, Ambrose Oruni, Catherine Maiteki-Sebuguzi, Grant Dorsey, Jimmy Opigo, Adoke Yeka, Agaba Katureebe, Mary Kyohere, Janet Hemingway, Moses R. Kamya, Martin J. Donnelly

**Affiliations:** 1 Liverpool School of Tropical Medicine, Pembroke Place, Liverpool, L3 5QA UK; 2 Infectious Diseases Research Collaboration, 2C Nakasero Hill Road, Kampala, Uganda; 3 London School of Hygiene & Tropical Medicine, Keppel Street, London, WC1E 7HT UK; 4 National Malaria Control Programme, Uganda Ministry of Health, Kampala, Uganda; 5 University of California, San Francisco, San Francisco, CA 94110 USA

**Keywords:** Malaria, Long-lasting insecticidal nets (LLINs), Piperonyl butoxide (PBO), Uganda, Cluster-randomised trial, Vector control, Insecticide resistance

## Abstract

**Background:**

Long-lasting insecticidal nets (LLINs) are the principal tool for malaria control in Africa and are presently treated with a single class of insecticide; however, increasing levels of insecticide resistance threaten their success. In response to this threat nets have been developed that incorporate the synergist, piperonyl butoxide (PBO), which inhibits the activity of cytochrome P450s which is one main mechanisms of insecticide resistance, allowing resistance to pyrethroids to be reversed. However, data on the value and cost effectiveness of these nets is lacking. A large-scale cluster randomised trial of conventional LLINs and PBO-LLINs was conducted in Uganda in 104 health sub-districts (HSDs) in 2017–2019. Prior to the mass distribution of LLINs, a baseline entomological survey was carried out, the results of which are reported herein. Ten households from each HSD were randomly selected for entomological surveillance at baseline which included household mosquito collections.

**Results:**

Prior to LLIN distribution entomological collections were carried out in 1029 houses across the 104 HSDs. *Anopheles gambiae* (*s.l.*) was the principal vector in all but 9 of the 71 HSDs that yielded vector species. Molecular analysis found *An. gambiae* (*s.s.*) to be the predominant vector collected. *Plasmodium falciparum* was detected in 5.5% of *An. gambiae* (*s.s.*) and in 4.0% of *An. funestus* (*s.s.*) examined. Infection rates of other plasmodium species (*P. vivax*, *P. ovale and P. malariae*) were lower with infection rates of 1.2% and 1.7% for *An. gambiae* (*s.s.*) and *An. funestus* (*s.s.*), respectively. The knockdown resistance (*kdr)* mutation *Vgsc*-L1014S was found at very high frequency in *An. gambiae* (*s.s.*) with the *Vgsc*-L1014F mutation at low frequency and the wild-type allele virtually absent. In *An. arabiensis* the wild-type allele was predominant. The resistance-associated alleles, *Cyp4j5*-L43F and *Coeae1d* were found at moderate frequencies which varied across the study site. *Vgsc*-N1575Y mutation was not found in any samples examined.

**Conclusions:**

No significant differences between planned intervention arms was observed in vector densities, sporozoite infection rate or insecticide resistance marker frequency across the study site prior to the distribution of LLINs. Very high levels of *kdr* resistance were observed in all areas; however, the resistance-associated markers *Cyp4j5*-L43F and *Coeae1d* were found at varying frequencies across the study site which may have implications for the effectiveness of standard LLINs.

*Trial registration* This study is registered with ISRCTN, ISRCTN17516395. Registered 14 February 2017, http://www.isrctn.com/ISRCTN17516395

**Electronic supplementary material:**

The online version of this article (10.1186/s13071-019-3353-7) contains supplementary material, which is available to authorized users.

## Background

The burden of malaria in sub-Saharan Africa has reduced markedly in the last 20 years. Malaria control has been focused on scaling up of insecticide treated net coverage (ITNs), indoor residual spraying (IRS), and treatment with artemisinin-based combination therapy (ACT) which together have reduced the incidence of clinical disease by 40% [1]. Increased coverage of vector control is estimated to have been the major driver behind the decline in malaria mortality between 2000–2015 [1, 2]. Long-lasting insecticidal nets (LLINs) are currently the principal tool for malaria control in Africa and are presently treated with a single class of insecticide; however, there is growing concern that the rapid spread of resistance to pyrethroids, could render LLINs ineffective [3, 4]. Moreover, the most recent data from 2016, suggest that after years of falling annual malaria mortality, advances may have stalled in Africa. There are therefore justified concerns about the emergence and spread of insecticide resistance and the impact this may have on the continued effectiveness of insecticide-based interventions [2, 3, 5, 6]. A recent WHO co-ordinated trial across a range of transmission settings in five countries concluded that users of LLINs still benefited from reduced rates of infection and morbidity even in areas where vectors show high levels of resistance to pyrethroids [5]. This earlier study was unable to determine whether the community effect had been lost, and as modelling data suggest increasing resistance will adversely affect malaria vector control [4] there is a pressing need for improved anti-vector interventions. One promising intervention is the development of LLIN technology which incorporates the synergist, piperonyl butoxide (PBO), which is intended to overcome one of the most important insecticide resistance mechanisms circulating in malaria vector populations. PBO inhibits the activity of cytochrome P450s and thus could reverse resistance to pyrethroids caused by these enzymes. Gene expression studies have demonstrated that P450s are differentially expressed in numerous populations of the primary African malaria vectors (*Anopheles gambiae*, *An. funestus* and *An. arabiensis*) [7–10]. Additional studies show that these overexpressed P450s are capable of metabolising pyrethroids and conferring elevated levels of insecticide resistance when expressed transgenically [7, 9, 11].

Resistance to pyrethroids in malaria vectors is widespread and intense in Uganda [6, 12, 13], and has been implicated in the limited epidemiological impact of both IRS- [14] and LLIN-based control campaigns [15]. P450-associated resistance has been observed in four districts in the East and North of the country [13] and a DNA diagnostic has been developed for a non-synonymous change in the p450, *Cyp4j5*, which has been reproducibly associated with pyrethroid resistance in several sites in Uganda and western Kenya [11]. Uganda is therefore an appropriate location to test whether combination PBO-LLINs are more effective than conventional LLINs.

**Fig. 1:**
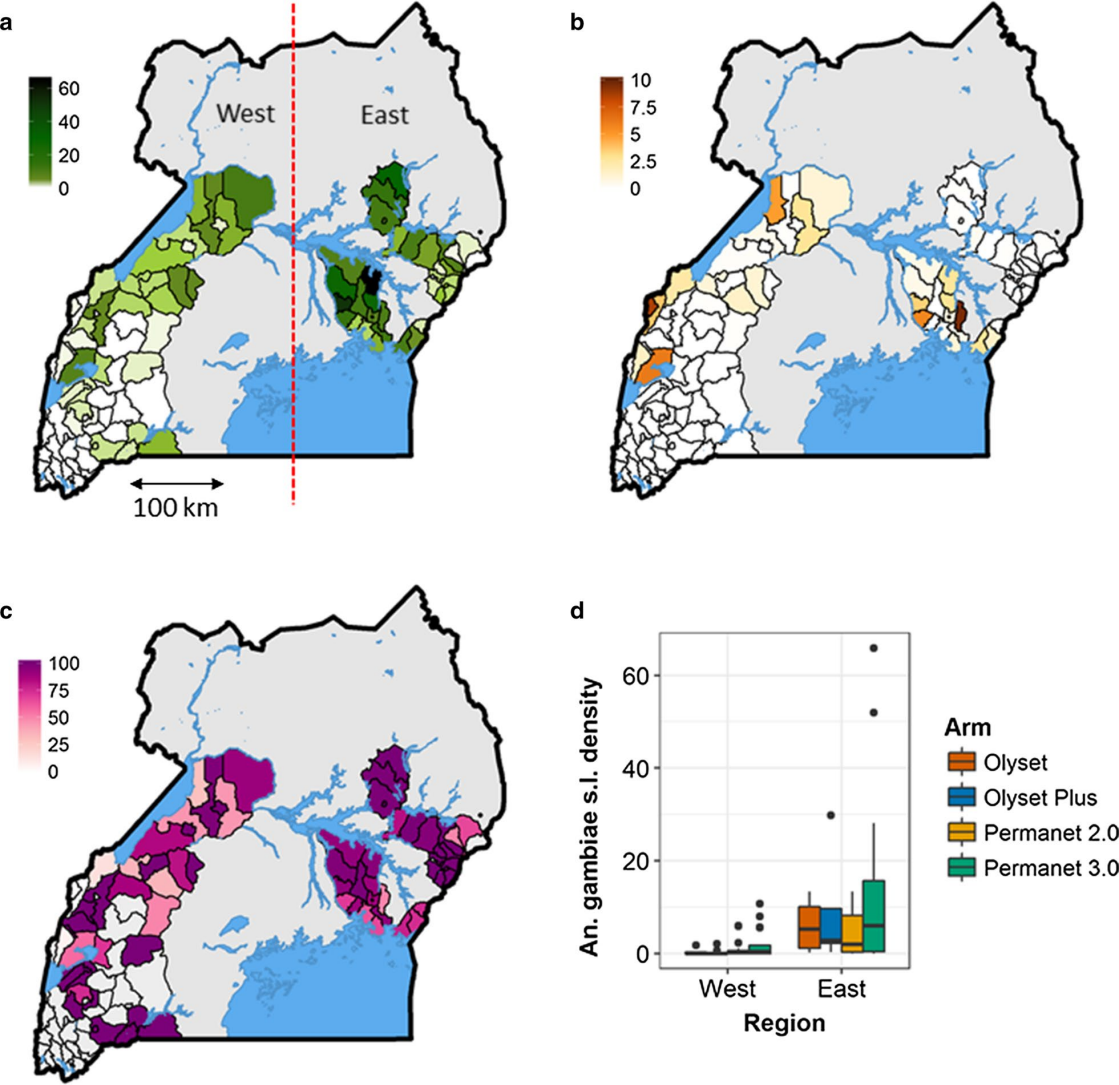
Mean mosquito density per house for *Anopheles gambiae* (*s.l.*) (**a**) and *Anopheles funestus* (*s.l.*) (**b**). **c** Relative proportion of *Anopheles gambiae* (*s.l.*), to total anophelines. **d** Box whisker plots illustrating the density of female *An. gambiae* (*s.l.*) by arm and region

A cross-sectional community survey was conducted in Uganda in 2017–2018 in 104 health sub-districts (HSDs) (48 districts) at baseline, prior to the mass distribution of LLINs by the Ministry of Health in 2017 and 2018 (Staedke et al. unpublished data). This mass distribution of nets was undertaken as a cluster randomised trial of conventional LLINs (Permannet 2.0 and Olyset) and PBO-LLINs (Permanent 3.0 and Olyset Plus) with the four different net types constituting the four arms of the study (registered trial ISRCTN 17516395). Of the 104 HSDs included in the study, 38 were located in the eastern region and the remaining 66 in the western region (Fig. 1a). The study region encompasses approximately half the total area of Uganda and includes a wide range of epidemiological settings with varying levels of malaria transmission and insecticide resistance [15, 16]. Prior to the survey, 50 households were randomly selected in each HSD for epidemiological surveys and a subset of 10 of these were selected at random for entomological surveillance which included household mosquito collections using mechanical aspirators and a questionnaire focusing on vector control at the household level. Full details of the epidemiological survey are presented in (Staedke et al. unpublished data). In this paper we present results from the entomological surveys.

In brief, mosquitoes were identified phenotypically to genus and a subset of *Anopheles* mosquitoes were identified to species and screened for malaria infection using molecular methods. Insecticide resistance of the primary malaria vector, *An. gambiae* (*s.s.*), was assessed by screening mosquitoes for the genetic signatures of insecticide resistance rather than traditional phenotypic methods. Previous studies have shown that resistance measured by phenotypic methods is not predicative of LLIN failure [5] and this approach can only detect large changes in resistance levels within a population. High levels of pyrethroid resistance across Uganda have been previously observed [6, 12, 13, 17], meaning that phenotypic screening may not be sensitive enough to detect small differences in resistance level temporally and geographically. Moreover, phenotypic assays are logistically difficult to perform on a large scale, and maybe unreliable if testing conditions are not rigorously controlled across all sites. Molecular screening of resistance-associated markers allows small changes in resistance to be observed and permits detailed geographical mapping of resistance across all 104 health sub-districts. Previous work in Uganda and Kenya has shown that several mutations in the P450, *Cyp4j5*, and the carboxylesterase gene, *Coeae1d*, are reliably associated with pyrethroid resistance and can be used as markers of resistance in this location [11]. These and other insecticide resistance-associated variants were genotyped in the primary malaria vector, *An. gambiae* (*s.s.*). Together these data (i) provide a comprehensive picture of malaria vector and insecticide resistance distribution across a broad swathe of Uganda; and (ii) enable us to determine if there were significant differences in vector distribution and insecticide resistance between planned intervention arms before the mass distribution of LLINs.

## Methods

### Household selection

A detailed sampling schema is given in (Staedke et al., unpublished data). In brief, a sample of 50 households from each of the 104 clusters was randomly selected using a two-stage cluster sampling procedure (Staedke et al., unpublished data). The randomization process was stratified by region, with 66 clusters in the western region, and 38 clusters in the eastern region (Fig. 1a), because whilst insecticide resistance is relatively well characterised in eastern Uganda comparatively little is known about patterns in the west. Enumeration areas (EAs) identified in the 2014 national census by the Uganda Bureau of Statistics formed the primary sampling unit [18]. Ten EAs were randomly selected in each of the 104 HSDs using probability proportionate to size (PPS) sampling. Households within each selected enumeration area were mapped and enumerated by the study team and a randomized list of households was produced in each EA. Households were approached for inclusion into the study by the study team until five households that had at least one child aged between 2–10 years present had been enrolled [19]. From the five households enrolled into the community survey in each EA, one household was randomly selected for enrolment into the entomological survey. Therefore, up to ten households were surveyed from each of the 104 HSDs included in the study, giving a maximum of 1040 households for entomological surveillance. Mosquito collections were made in the early morning and a questionnaire completed. Consent was obtained the previous day. Entomological data were collected on paper forms and later transferred to a Microsoft Access database. Internal consistency checks were carried out.

### Collections

Mosquitoes were collected from houses using mechanical (‘Prokopack’) aspirators [20]. Household collections were carried out by one person per house for a duration of ten minutes before 10:00 h. All mosquitoes collected were counted and morphologically identified to genus. *Anopheles* mosquitoes were identified to species group [21] and blood-fed status recorded. *Anopheles* mosquitoes were stored individually in pierced 0.2 ml tubes and sealed in ziplock bags containing silica gel. Up to 50 *Anopheles gambiae* (*s.l.*) and 50 *An. funestus* (*s.l.*) mosquitoes per HSD were selected for molecular analysis. DNA extractions were carried out on the head and thorax using Nexttec Biotechnologie DNA extraction plates (Nexttec Biotechnologie GmbH, Hilgertshausen, Germany) according to the manufacturer’s instructions.

### Molecular analysis

Mosquitoes identified morphologically as *An. gambiae* (*s.l.*) were identified to species level by SINE PCR [22]. Mosquitoes identified phenotypically as *An. funestus* (*s.l.*) were identified to species by PCR of the ITS2 region on the rDNA [23]. Presence of the 2La chromosome inversion in *An. gambiae* (*s.l.*) specimens was assessed by PCR [24]. Sporozoite rates in *An. gambiae* (*s.l.*) and *An. funestus* (*s.l.*) were calculated using the Taqman assay for detection of *Plasmodium falciparum*, *P. vivax*, *P. ovale* and *P. malariae* [25]. *Anopheles gambiae* (*s.l.*), the dominant vector group, were screened for a number of mutations known to be associated with pyrethroid resistance. The *Vgsc*-L1014F and *Vgsc*-L1014S mutations were assayed using the TaqMan assay [26] or LNA-kdr assay [27]. Screening for the *Vgsc*-N1575Y, *Cyp4J5*-L43F and *Coeae1d* mutations used TaqMan assays following standard protocols [11, 28]. All Taqman and LNA assays were analysed using AriaMX V1.5 or MXPro software and the ΔR threshold adjusted manually for each dye, if necessary. Results for “ΔR last”, the final baseline-corrected fluorescence reading as measured in the last cycle, were then exported into Microsoft Excel for analysis and the genotype at each locus determined.

Data from household mosquito collections and questionnaires was compiled in Microsoft Access. Molecular data was compiled in Microsoft Excel. All data analyses were carried out using R statistical software version 3.3.1. Analysis of mosquito density used Generalized Linear Mixed Effect Models (GLMMs) using a Type 2 Negative Binomial distribution with model fit determined by Akaike information criterion (AIC) to account for repeated measures (up to ten households per HSD). Analysis of frequency data (sporozoite infections and molecular markers) used General Linear Models with logit link function for a binomial dependent variable.

## Results

Entomological collections were carried out from 1029 houses. A total of 4703 female anopheline mosquitoes were collected of which 4009 were identified morphologically as *An. gambiae* (*s.l.*) and 694 as *An. funestus* (*s.l.*) (Fig. 1a, b). Female individuals were collected in 71 of 104 HSDs and in all but 9 of the 71 HSDs *An. gambiae* (*s.l.*) was predominant (Fig. 1c). The density of female *An. gambiae* (*s.l.*) (Fig. 1d) and *An. funestus* (*s.l.*) (Additional file 1: Figure S1) was analysed as a function of planned net distribution arm with HSD as random effect using GLMM based on a Type 2 Negative Binomial Model. The best-fit model for both species, was determined by AIC, a method which allows the relative fit of different models to be estimated for a dataset, with lower values indicating a better relative fit than higher values. The best-fit model was found to include region (East *vs* West) but did not include planned intervention arm showing that there was no significant difference in the densities of either *An. gambiae* (*s.l.*) or *An. funestus* (*s.l.*) by planned intervention arm (Table 1).

**Table 1:** Mosquito density analysed as a function of net distribution arm and region with HSD as random effect using a logistic GLMM binomial model

Response variable	Fixed effects	Random effects	AIC	dAIC	*df* lost
*An. gambiae* (*s.l.*) females	Region	HSD	2597.8	0	4
	Region + Arm	HSD	2598.8	1.0	7
	Arm	HSD	2632.1	34.3	6
	−	HSD	2634.5	36.7	3
*An. funestus* (*s.l.*) females	Region	HSD	1111.7	0	4
	Region + Arm	HSD	1115.8	4.1	7
	−	HSD	1116.3	4.6	3
	Arm	HSD	1118.9	7.2	6

*Abbreviation*: df, degrees of freedom

Heads and thoraces from 1942 *Anopheles* mosquitoes were separated from abdomens, and DNA extracted for molecular analysis. Of these, 1368 were *An. gambiae* (*s.s.*), 88 *An. arabiensis*, 470 *An. funestus* (*s.s.*) and 1 *An. parensis.* Morphological identification was accurate for 86% of mosquitoes identified phenotypically as *An. funestus* (*s.l.*) and 94% of mosquitoes identified as *An. gambiae* (*s.l.*)

Molecular identification was not possible for 15 specimens using standard PCR for *An. gambiae* and *An. funestus* species groups. Successful amplification and sequencing of the mitochondrial cytochrome *c* oxidase subunit 1 (mtDNA *cox*1) gene (using primers TL2-N-3014 and CI-J-2183) [29] was achieved for 11 out of these 15 specimens. A BLAST search against the NCBI database [30] was carried out to identify similarity to known anopheline sequences. The observed percentage identity ranged between 92–99%, with a query coverage of 96–100%. The sequences of the top matches were collated, and redundant sequences removed, before alignment using multiple sequence alignment by MUSCLE [31] and a phylogenetic tree constructed using PHYML algorithm [32] (Additional file 2: Figure S2). Three samples were clustered in the *An. gambiae* (*s.l.*) group, and three within the *An. funestus* (*s.l.*) group. Two specimens, one of which was positive for *P. falciparum*, clustered with *An. pharoensis* (percentage identity 93%); one sample was most likely *An. rufipes* (percentage identity of 95%), whilst two specimens were of uncertain status clustering with *Anopheles minimus* and *Anopheles culicifacies*, both vectors not found in sub-Saharan Africa.

**Fig. 2:**
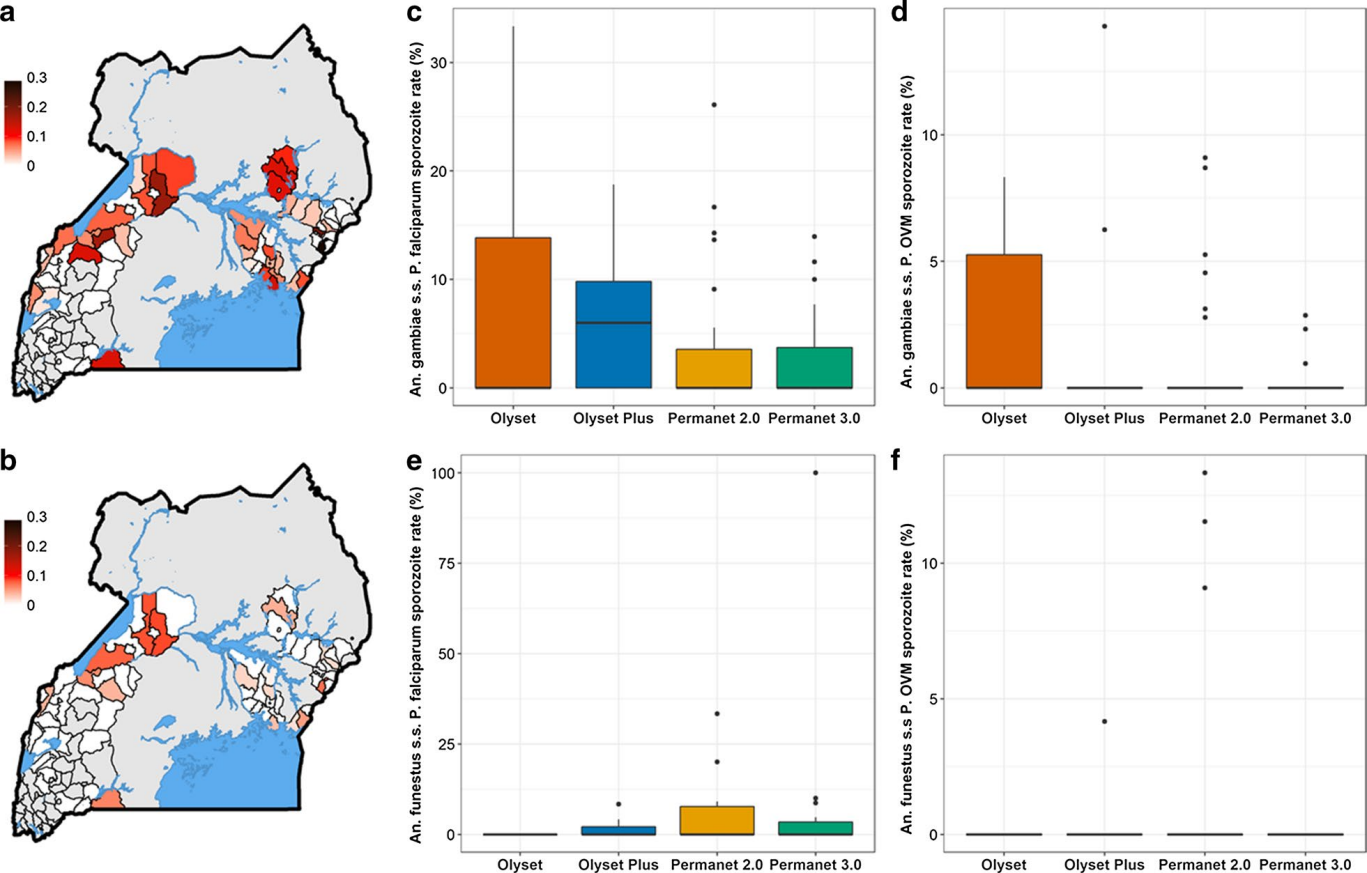
Sporozoite infection rates. **a** *P. falciparum* sporozoite infection rate in *Anopheles.* **b** Sporozoite infection rate for combined *P. vivax*, *P. ovale* and *P. malariae* in *Anopheles*. **c** *P. falciparum* sporozoite infection rate in *An. gambiae* (*s.s.*) by net distribution arm. **d** Sporozoite infection rate for the combined *P. vivax*, *P. ovale* and *P. malariae* (P.OVM) in *An. gambiae* (*s.s.*) by net distribution arm. **e** *P. falciparum* sporozoite infection rate in *An. funestus* (*s.s.*) by net distribution arm. **f** Sporozoite infection rate for combined *P. vivax*, *P. ovale* and *P. malariae* (P. OVM) in *An. funestus* (*s.s.*) by net distribution arm

Sporozoite infection rates varied across the study site (Fig. 2a, b, Additional file 3: Figure S3). *Plasmodium falciparum* was detected in 5.5% of *An. gambiae* (*s.s.*) and in 4.0% of *An. funestus* (*s.s.*) examined. No *Plasmodium* spp. were observed in the limited sample of *An. arabiensis.* Infection rates of other *Plasmodium* species (*P. vivax*, *P. ovale* and *P. malariae*) were lower with infection rates of 1.2% and 1.7% for *An. gambiae* (*s.s.*) and *An. funestus* (*s.s.*), respectively. *Plasmodium* infection rates in *An. gambiae* (*s.s.*) (Fig. 2c, d) and *An. funestus* (*s.s.*) (Fig. 2e, f) were analysed as a function of net distribution arm with HSD as random effect using a logistic GLMM Binomial Model. The best-fit model, as determined by AIC, did not include intervention arm showing that there was no significant difference in infection rates by planned intervention arm.

**Fig. 3:**
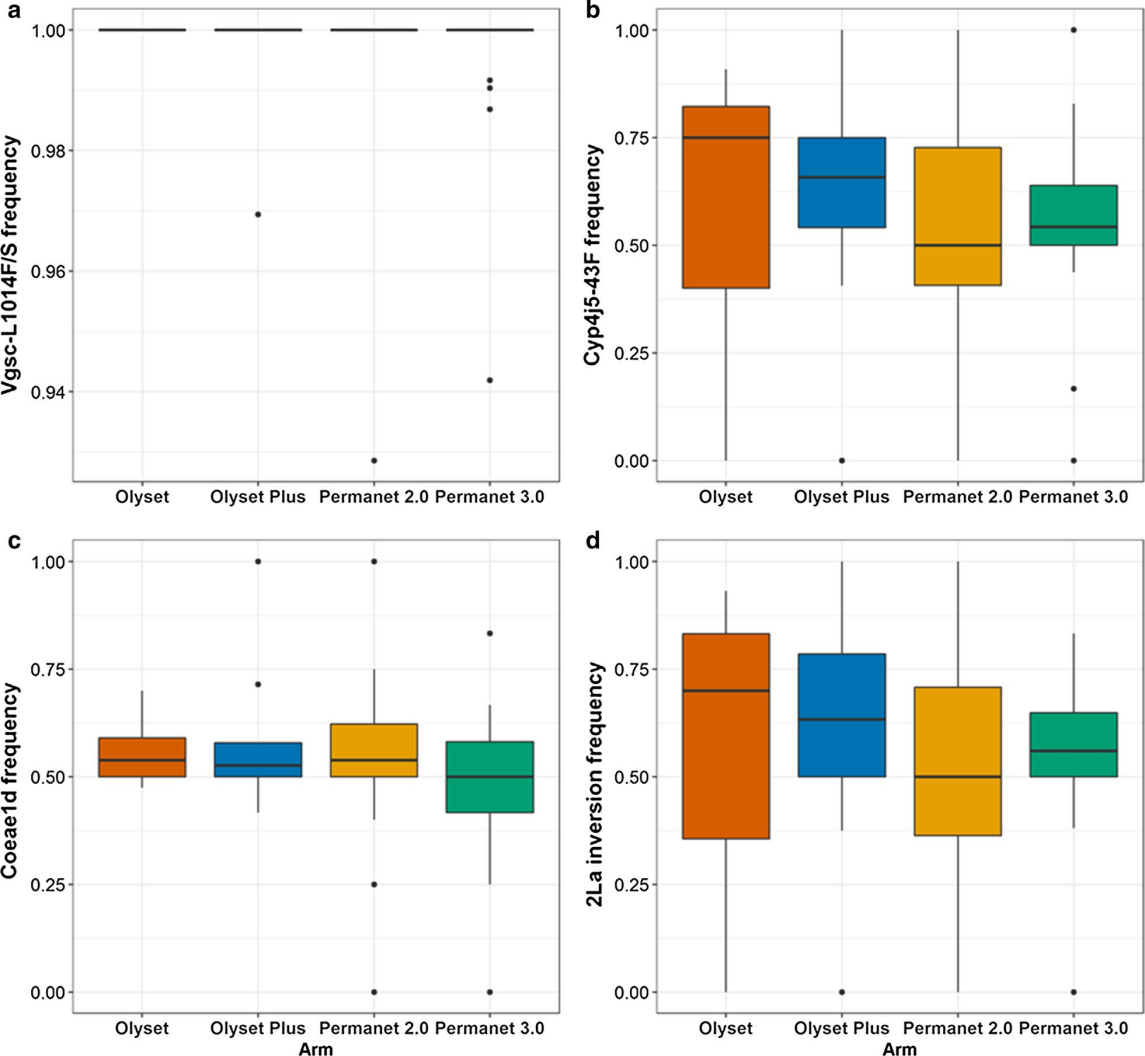
Resistance and polytene chromosome allele frequencies in *Anopheles gambiae* (*s.s.*) by net distribution arm. **a** *Vgsc* 1014F/S. **b** *Cyp4j5*-L43F. **c** *Coeae1d*. **d** 2La inversion

**Fig. 4:**
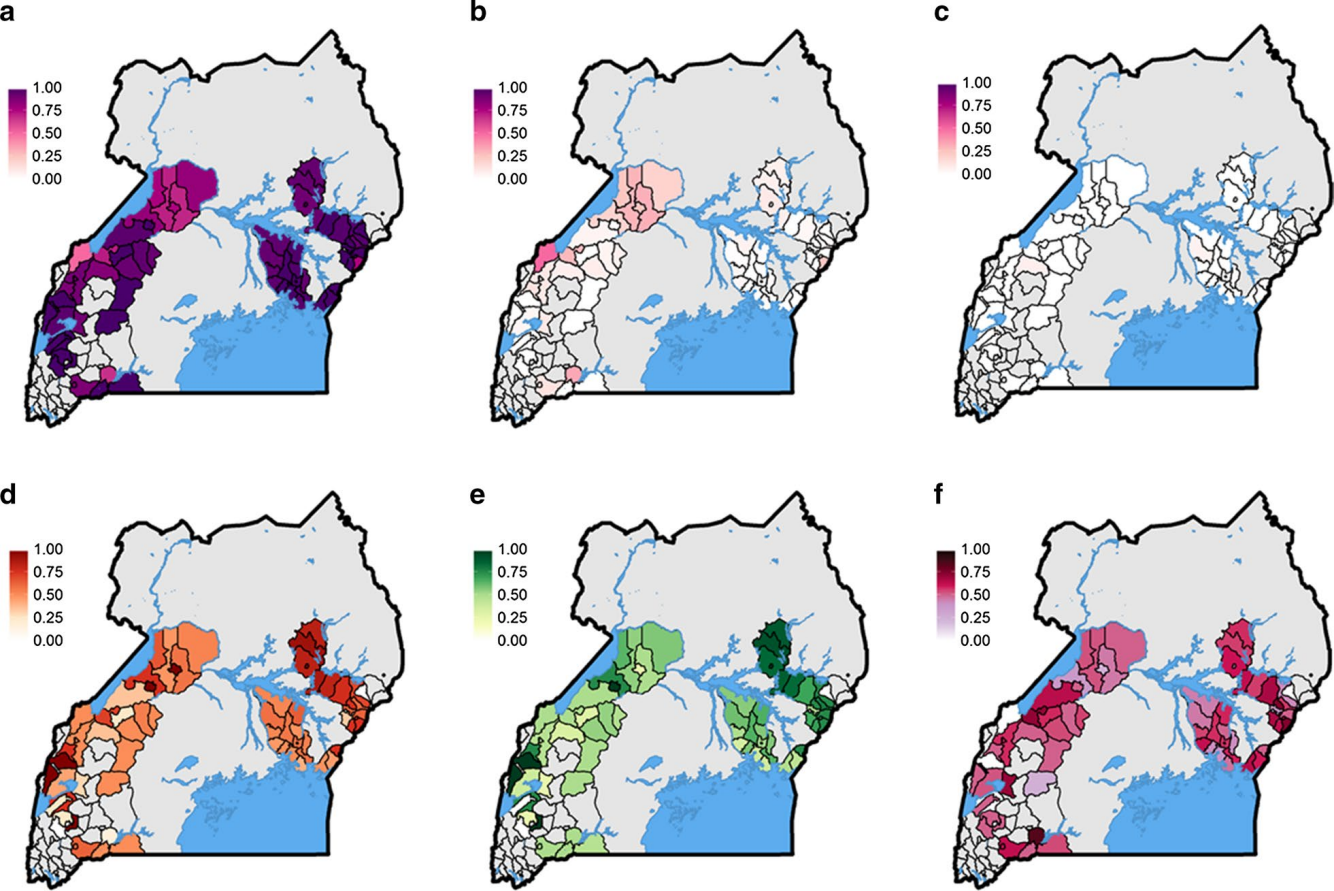
Resistance and polytene chromosome allele frequencies in *Anopheles gambiae* (*s.s.*). **a** *Vgsc*-1014S. **b** *Vgsc*-1014F. **c** *Vgsc*-1014L. **d** *Cyp4j5*-L43F. **e** 2La inversion. **f** *Coeae1d*

Resistance screening of the primary vector group, *An. gambiae* (*s.l.*) was carried out, and health sub-district specific resistance marker frequency data were generated for the resistance variants *Vgsc*-L1014S, *Vgsc*-L1014F, *Vgsc*-N1575Y, *Cyp4j5*-L43F and *Coeae1d* mutations, using PCR based approaches. The *kdr* mutations, *Vgsc*-L1014S and *Vgsc*-L1014F, that cause alterations to the target site of the voltage-gated sodium channel and are associated with resistance to pyrethroids and DTT, were both detected in our sample. In *An. gambiae* (*s.s.*) the L1014S was at a frequency of 0.94, whilst *Vgsc*-L1014F was detected at a frequency 0.06 (Figs. 3a, 4a-c, Additional file 4: Figure S4). The wild-type allele was detected at extremely low levels (< 0.01). In *An. arabiensis* the wild-type allele was detected at a frequency of 0.96, whilst resistance-associated alleles were detected at a frequency of 0.01 and 0.03 for L1014S and L1014F respectively.

Samples were screened for the *Vgsc*-N1575Y mutation, which is also located in the voltage-gated sodium channel and is known to have a synergistic effect on pyrethroid and DDT resistance when combined with the *Vgsc*-L1014F mutation [28]. The mutation, which has been found across West and Central Africa, including the neighbouring country of DRC in *An. gambiae* (*s.s.*), *An. coluzzii* and *An. arabiensis* [27, 28, 33], was not found in any of the specimens examined. This is perhaps unsurprising given the low frequency of the L1014F mutation in the population. The pyrethroid metabolic resistance-associated variant, *Cyp4j5*-L43F [11], located within a p450 gene in the 2La inversion was found at a frequency of 0.60 in *An. gambiae* (*s.s.*) (Figs. 3b, 4d, Additional file 4: Figure S4). The *Coeae1d* resistance-associated allele [11] located in a carboxylesterase gene and highly associated with pyrethroid resistance in East African *An. gambiae* populations, was found at a frequency of 0.54 in *An. gambiae* (*s.s.*) (Figs. 3c, 4f, Additional file 4: Figure S4). The 2La chromosome inversion, associated with adaptation to aridity and humidity [34, 35], biting and resting behaviour [36], and increased susceptibility to *Plasmodium* [37] was found at a frequency of 0.60 in *An. gambiae* (*s.s.*) (Figs. 3d, 4e, Additional file 4: Figure S4).

Resistance marker frequencies for *Anopheles gambiae* (*s.s.*) were analysed as a function of net distribution arm and region (East *vs* West) using GLM. No evidence for a significant difference in resistance marker frequency by planned intervention arm or region was observed (Table 2 and Additional file 5: Table S1).

**Table 2:** Resistance and polytene chromosome allele frequencies in *Anopheles gambiae* (*s.s.*) analysed as a function of net distribution arm using a generalized linear model

Response variable	*df*	Deviance	Residual *df*	Residual deviance	*P*
Vgsc-1014	3	0.04546	64	1.0067	0.9975
Cyp4j5-43F	3	0.12217	64	22.340	0.9891
Coeae1d	3	0.61043	64	11.074	0.8940
X2La	3	0.20198	64	21.586	0.9773

*Abbreviation*: df, degrees of freedom

## Discussion

This study provides a comprehensive description of the distribution of major malaria vector species in Uganda, reporting data from 48 of 121 administrative districts. *Anopheles gambiae* (*s.s.*) was the dominant endophilic vector across the majority of sites in Uganda with *An. funestus* and *An. arabiensis* generally observed at lower densities. *Plasmodium falciparum* infection rates of 5.5% in *An. gambiae* (*s.s.*) and 4.0% in *An. funestus* (*s.s.*) were higher than previously observed in Uganda [38]. This may partially reflect differences in the assay methods used in this work compared to the previous study, ELISA *vs* Taqman, with the latter having increased sensitivity [25]. Whilst the Prokopak-based collection method precludes the estimation of entomological inoculation rates, these data suggest high rates of transmission across Uganda despite widespread conventional LLIN use [19, 39]. These data corroborate early work which has revealed a relatively limited impact of both DDT and pyrethroid based vector control on epidemiological indices [14, 15] thereby highlighting the need for alternative vector control technologies.

Three additional potential vector species were detected: *An. parensis*, *An. pharoensis* and *An. rufipes*. All three species have been recorded in Uganda and in parts of their range they have been found to be sporozoite-positive [40]. It is possible that these species may be malaria vectors in some of the study locations, and whilst the single specimen of *An. pharoensis* was positive for *P. falciparum* DNA, these species were captured at very low densities in our indoor-collections. It is possible that these species might have a role in the maintenance of residual transmission and in the transmission of filarial and viral infections [40].

Three known pyrethroid resistance-associated variants were widely distributed across the country. The knockdown resistance mutations (*Vgsc*-1014F/S) were near or at fixation in *An. gambiae* (*s.s.*) populations as expected from earlier studies [6, 11, 41, 42]. The presence of the Vgsc-1014F mutation has been reported sporadically in the region [41, 43] and despite evidence that it may confer higher rates of resistance to pyrethroids that the Vgsc-1014S mutation (reviewed in [44]) there is to date no conclusive evidence of allelic replacement. The two metabolic resistance-associated variants *Cyp4j5*-L43F and *Coeae1d* were found across the country at intermediate frequencies. The importance of these mutations on the effectiveness of PBO-LLINs in the field is not yet fully understood however it is hoped that by screening for these resistance markers at six monthly intervals (6, 12 and 18 months post-baseline) it will be possible to track the differential impact of the interventions in areas of differing insecticide resistance.

## Conclusions

Importantly for the purposes of the cluster randomized trial there were no significant differences observed between planned the intervention arms at baseline for any of the key variables; vector density, *Plasmodium* infection rates or insecticide resistance marker frequency. In the principal vector, *An. gambiae* (*s.s.*), extreme levels of *kdr* resistance were observed in all areas with *Vgsc-*L1014S predominating. The resistance-associated markers, *Cyp4j5*-L43F and *Coeae1d* were found at differing frequencies across the study site which may have consequences for the effectiveness of standard and PBO impregnated LLINs.

## Additional files

**Additional file 1: Figure S1.** The density of female *An. funestus* (*s.l.*) was analysed as a function of net distribution arm with HSD as random effect using GLMM based on a Type 2 negative Binomial Model. The best-fit model, as determined by AIC, did not include planned intervention arm showing that there was no significant difference in the densities of *An. funestus* (*s.l.*) by intervention.

**Additional file 2: Figure S2.** Phylogenetic tree constructed from mitochondrial *cox*1 gene sequence of the unknown mosquito samples (in red boxes) were compared with known sequences of major anopheline vectors from the NCBI database. A mid-rooted phylogenetic tree was plotted using PHYML (Maximum Likelihood relationship) algorithm, following multiple sequence alignment by MUSCLE. The black dots are the tree nodes which represent a common ancestor. The figures show the branch length which represents the amount of change in-terms of mutations that has occurred with time between members.

**Additional file 3: Figure S3.** Sporozoite infection rates by sub region. **a** *P. falciparum* sporozoite infection rate in *An. gambiae* (*s.s.*). **b** Combined sporozoite infection rate for *P. vivax*, *P. ovale* and *P. malariae* in *An. gambiae* (*s.s.*). **c** *P. falciparum* sporozoite infection rate in *An. funestus* (*s.s.*). **d** Combined sporozoite infection rate for *P. vivax*, *P. ovale* and *P.* *malariae* in *An. funestus* (*s.s.*).

**Additional file 4: Figure S4.** Resistance and polytene chromosome allele frequencies in *Anopheles gambiae* (*s.s.*) by sub-region. **a** *Vgsc* 1014F/S. **b** *Cyp4j5*-L43F. **c** *Coeae1d*. **d** 2La inversion.

**Additional file 5: Table S1.** Resistance and polytene chromosome allele frequencies in *Anopheles gambiae* (*s.s.*) analysed as a function of sub-region using a generalized linear model.
