# Supplementary figures and images for: LLIN Evaluation in Uganda Project (LLINEUP): a cross-sectional survey of species diversity and insecticide resistance in 48 districts of Uganda
Source: Parasit Vectors. 2019 Mar 12;12:94. doi: 10.1186/s13071-019-3353-7 (PMC6417037; doi:10.1186/s13071-019-3353-7)

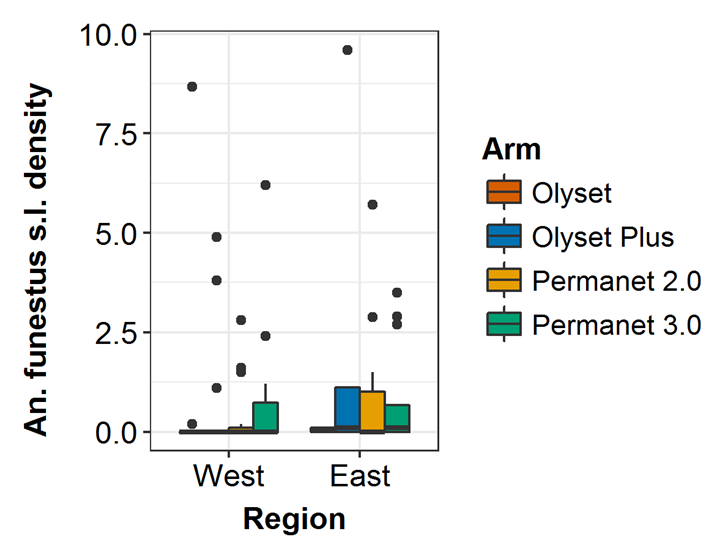

Supplement: Supplementary file 1 — Additional file 1: Figure S1. The density of female An. funestus (s.l.) was analysed as a function of net distribution arm with HSD as random effect using GLMM based on a Type 2 negative Binomial Model. The best-fit model, as determined by AIC, did not include planned intervention arm showing that there was no significant difference in the densities of An. funestus (s.l.) by intervention. [file 13071_2019_3353_MOESM1_ESM.tif]

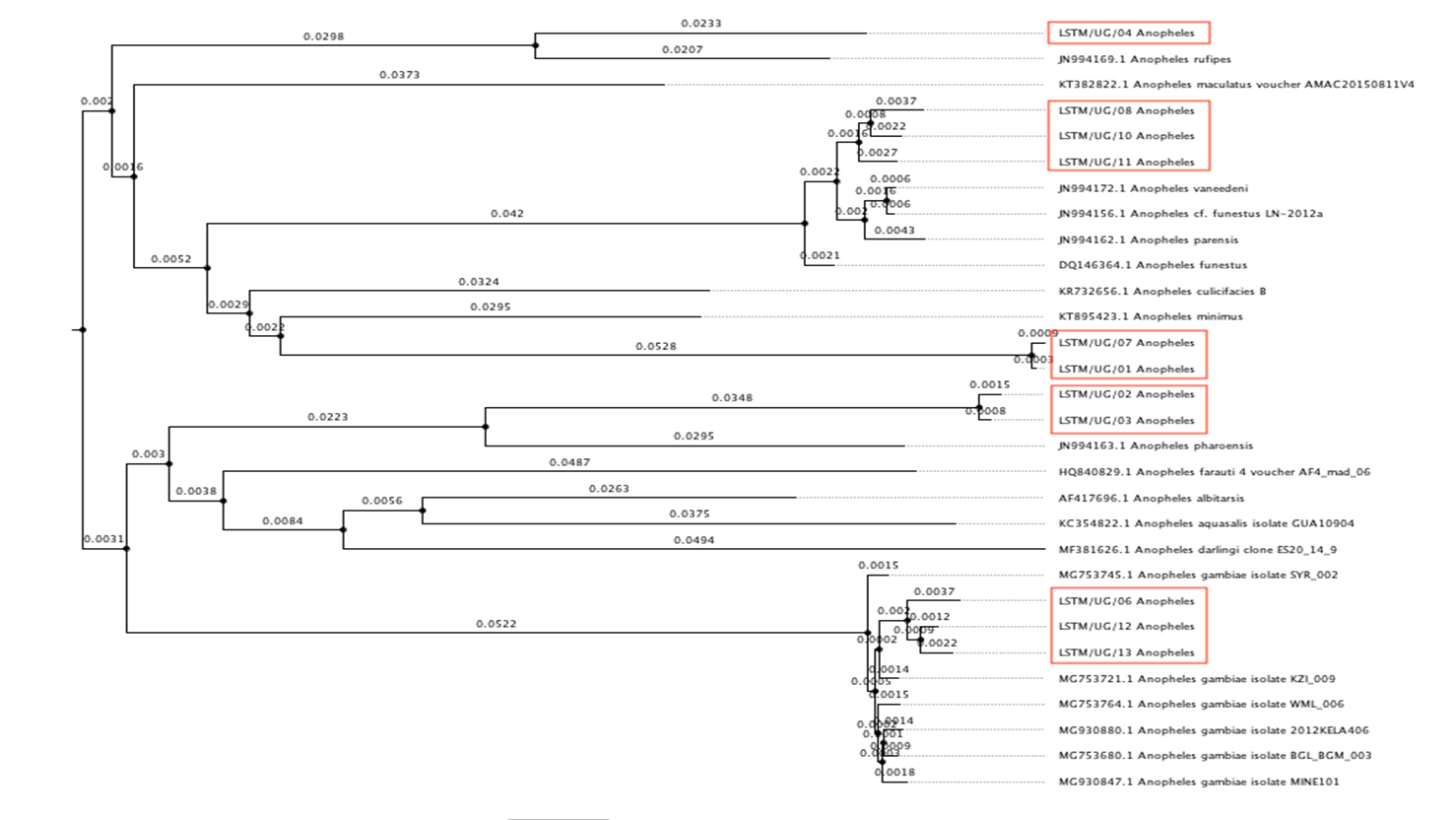

Supplement: Supplementary file 2 — Additional file 2: Figure S2. Phylogenetic tree constructed from mitochondrial cox1 gene sequence of the unknown mosquito samples (in red boxes) were compared with known sequences of major anopheline vectors from the NCBI database. A mid-rooted phylogenetic tree was plotted using PHYML (Maximum Likelihood relationship) algorithm, following multiple sequence alignment by MUSCLE. The black dots are the tree nodes which represent a common ancestor. The figures show the branch length which represents the amount of change in-terms of mutations that has occurred with time between members. [file 13071_2019_3353_MOESM2_ESM.tif]

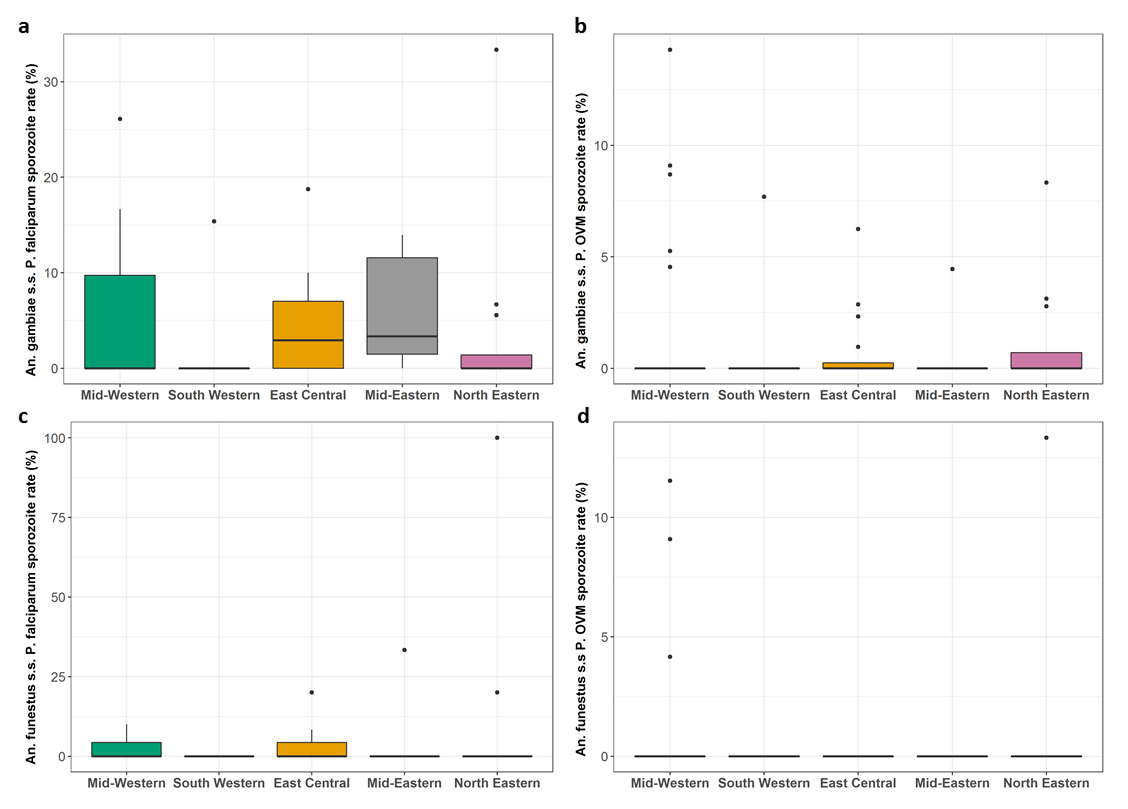

Supplement: Supplementary file 3 — Additional file 3: Figure S3. Sporozoite infection rates by sub region. a P. falciparum sporozoite infection rate in An. gambiae (s.s.). b Combined sporozoite infection rate for P. vivax, P. ovale and P. malariae in An. gambiae (s.s.). c P. falciparum sporozoite infection rate in An. funestus (s.s.). d Combined sporozoite infection rate for P. vivax, P. ovale and P. malariae in An. funestus (s.s.). [file 13071_2019_3353_MOESM3_ESM.tif]

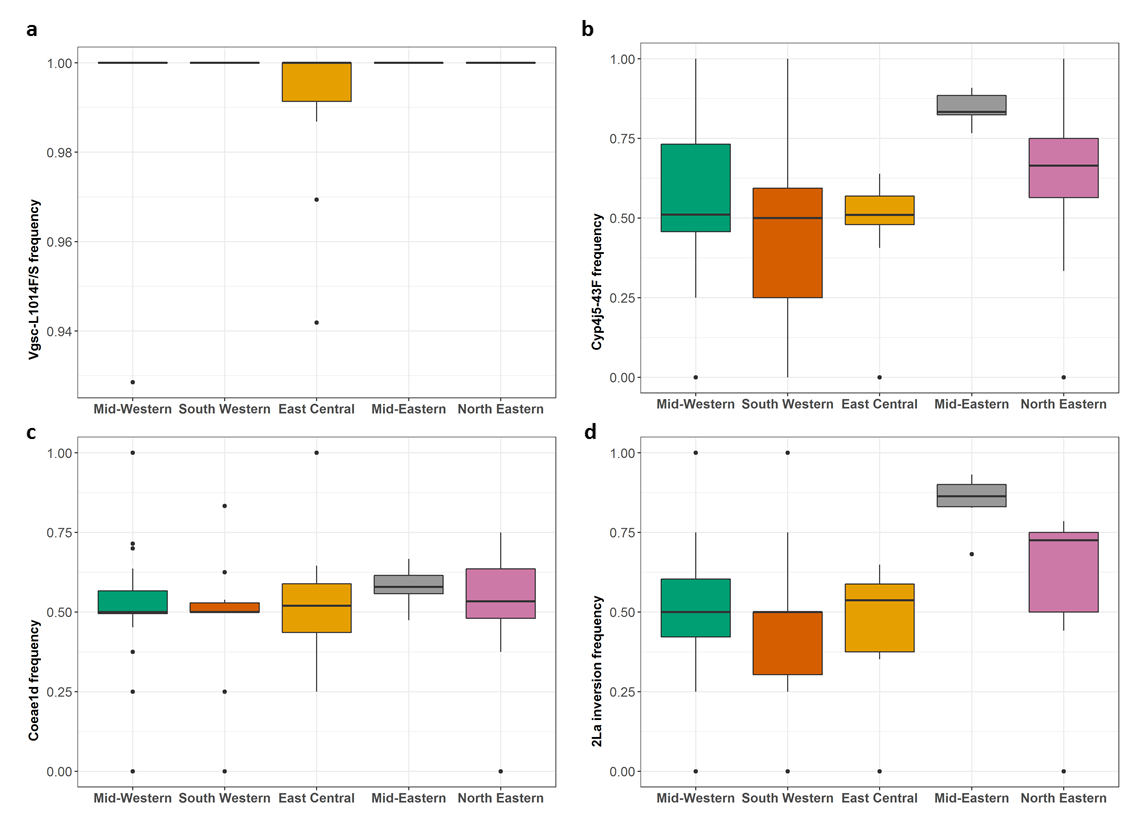

Supplement: Supplementary file 4 — Additional file 4: Figure S4. Resistance and polytene chromosome allele frequencies in Anopheles gambiae (s.s.) by sub-region. a Vgsc 1014F/S. b Cyp4j5-L43F. c Coeae1d. d 2La inversion. [file 13071_2019_3353_MOESM4_ESM.tif]
