# Supplementary material for: LLIN Evaluation in Uganda Project (LLINEUP): a cross-sectional survey of species diversity and insecticide resistance in 48 districts of Uganda
Source: Parasit Vectors. 2019 Mar 12;12:94. doi: 10.1186/s13071-019-3353-7 (PMC6417037; doi:10.1186/s13071-019-3353-7)
Supplement: Supplementary file 5 — Additional file 5: Table S1. Resistance and polytene chromosome allele frequencies in Anopheles gambiae (s.s.) analysed as a function of sub-region using a generalized linear model. [file 13071_2019_3353_MOESM5_ESM.docx]

**Additional file 5**: **Table S1.** Resistance and polytene chromosome allele frequencies in *Anopheles gambiae s.s.* analysed as a function of region using a generalized linear model

| Response variable | Df | Deviance | Residual Df | Residual  Deviance | P |
| --- | --- | --- | --- | --- | --- |
| Vgsc-1014 | 1 | 0.00974 | 66 | 1.0424 | 0.9214 |
| Cyp4j5-43F | 1 | 0.18696 | 66 | 22.279 | 0.6655 |
| Coeae1d | 1 | 0.14738 | 66 | 11.537 | 0.7011 |
| X2La | 1 | 0.47873 | 66 | 21.309 | 0.489 |
